# Congenital Sucrase–Isomaltase Deficiency: Same Mutation with Different Clinical Presentations

**DOI:** 10.5152/tjg.2024.23250

**Published:** 2024-04-01

**Authors:** Fatma İssi Irlayıcı, Burcu Güven, Murat Çakır

**Affiliations:** Department of Pediatric Gastroenterology, Karadeniz Technical University Faculty of Medicine, Trabzon, Turkey

**Keywords:** Congenital sucrase–isomaltase deficiency, chronic diarrhea, sacrosidase

## Abstract

**Background/Aims:**

Congenital sucrase–isomaltase deficiency is an autosomal recessive inherited disaccharidase deficiency characterized by chronic osmotic diarrhea. In this study, the genotype–phenotype relationships of close relatives of an index case with congenital sucrase–isomaltase deficiency were investigated.

**Materials and Methods:**

A 23-month-old female patient with a *sucrase–isomaltase *gene c.317G>A (p.C106Y) homozygous mutation was diagnosed as an index case and her pedigree analysis was performed subsequently. The family members with and without *sucrase–isomaltase *gene mutations were compared in terms of clinical symptoms.

**Results:**

The study included 109 cases [mean age ± SD: 22.6 ± 17.2 years (0.1-75 years), 61 males (56%)] of 130 family members of the index case. *Sucrase–isomaltase* gene c.317G>A (p.C106Y) heterozygous mutation was detected in 27 cases (24.7%); 14 (51.9%) were male and had a mean age of 23.2 ± 18.3 years. The most common complaints of 12 (44.4%) symptomatic patients with mutations were abdominal pain (37%), gas irritability (33.3%), bloating (22.2%), and foul-smelling stools (18.5%). Compared with the cases without mutation, a statistically significant difference was observed in the incidence of gas irritability, foul-smelling stool, ≥2 gastrointestinal symptoms, postprandial complaints, and food allergy (*P* = .005, *P* = .047, *P* = .049,* P* = .017, *P* = .021, respectively). Sacrosidase enzyme replacement was applied to 7 patients whose symptoms did not improve with dietary elimination. Clinical response was obtained after enzyme treatment.

**Conclusion:**

Despite its autosomal recessive inheritance, congenital sucrase–isomaltase deficiency can also be symptomatic in heterozygous individuals. Further studies are required to clarify the genotype–phenotype relationship and management of the disease.

**Main Points**

- Congenital sucrase–isomaltase deficiency is an autosomal recessive (AR) inherited disaccharidase deficiency.
- As a result of carbohydrate maldigestion, clinical symptoms include chronic osmotic diarrhea, bloating, and gas irritability.
- Although the disease follows a pattern of AR inheritance, clinical symptoms can also be observed in individuals with heterozygous mutations in the *sucrase–isomaltase* gene.
- The clinical presentations of patients with the same heterozygous mutation may vary. Factors that contribute to this variability are thought to be multifactorial in nature and include age, nutritional status, and enzyme activity level.
- Further studies are needed to evaluate the clinical management, indications for enzyme replacement therapy, and the genotype–phenotype relationship in the patients with heterozygous mutations.

## Introduction

Congenital sucrase–isomaltase deficiency (CSID) is an autosomal recessive (AR) disease characterized by chronic osmotic diarrhea with a mutation in the *sucrase–isomaltase (SI)* gene localized on chromosome 3 (3q25-26).^1^

It is classified as a rare genetic disease, with a prevalence of ~0.2% in North America and Europe. However, it can affect as much as 10% of the population in isolated populations like Greenland.^2,3^ With more than 40 mutations identified in recent years, it is considered that the disease may be more common than previously anticipated.^4,5^

The absence or deficiency of the sucrase–isomaltase enzyme leads to fermentation of unabsorbed carbohydrates in the distal small intestine and colon. As a result of this, the excessive production of gases such as hydrogen, methane, and hydrogen sulfide and short-chain fatty acids occur.^6^ Children may get admitted with more prominent symptoms (abdominal bloating, cramps, and osmotic diarrhea) since they have shorter small intestinal transit time and their colon has less capacity to absorb the increased luminal fluid.^7^ Failure to thrive, dehydration, and malnutrition may be observed in untreated cases. Adolescents and adults can receive a diagnosis of nonspecific diarrhea or diarrhea-dominant irritable bowel disease.^8^

The clinical presentation of the disease may vary depending on whether the *SI* gene mutations are homozygous or heterozygous, and on residual enzyme activity. Enzyme activity ranges from absent to decreased activity for sucrase and absent to normal for isomaltase. A significant reduction in maltase activity has been reported in most patients.^9^ Other factors that can affect the development of symptoms include the amount of sucrose and starch consumed in the diet and the age of the patient.

Recent studies have shown that patients with heterozygous genotype may also be symptomatic.^10^ Theoretically, in the presence of a heterozygous mutation, one of the alleles will be normal and contain sucrase–isomaltase molecules with 50% active digestion capacity. In this case, it is expected that the disaccharides will be digested and malabsorption symptoms will not occur. However, in some heterozygous cases, low sucrase–isomaltase enzyme levels have been reported to cause malabsorption. This is thought to be due to the single mutant allele having a regulatory effect on wild-type *SI* in the secretory pathway, in addition to its functional and biosynthetic function.^11^ Studies revealed that when the wild-type SI gene is affected, SI protein expression decreases, which is associated with higher frequency of symptoms.^12^ Another hypothesis concerns mosaic or heterogeneous expression of disaccharidases from enterocytes. Regardless of the change in the *SI* gene, enzyme expression may be decreased in different regions of the intestinal epithelium due to the decrease in carbohydrate digestive capacity.^13^

In this study, we aimed to perform genetic screening and symptom evaluation for CSID in family members identified by pedigree analysis of our index case with a homozygous pathogenic mutation in the *SI *gene. We think that the data obtained by demonstrating different clinical features, especially in heterozygous individuals with the same mutation, will contribute to the literature.

## Materials and Methods

This study was an observational, prospective, epidemiological genetic screening study.

### Index Case

The 23-month-old girl index case with consanguineous parents was the first child of the family, and there were recurrent hospital admissions due to excessive watery stools 7-8 times a day and abdominal distension starting at 6 months of age. The onset of her complaints was correlated with the transition to complementary feeding, triggered by foods containing sugar and starch, and did not regress with an amino acid-based formula. On physical examination, weight was 9700 g (3-10 percentile), height was 81 cm (25-50 percentile), and other system examination findings were normal except abdominal distension. Biochemical parameters, serum and specific immunoglobulin E, food panel, tissue transglutaminase immunoglobulin A (IgA), and stool tests were normal. The upper gastrointestinal (GI) endoscopic and histological findings were normal and the patient was prescribed a sucrose- and starch-restricted diet. Since her symptoms were partially improved with diet, genetic analysis was performed for CSID. Upon detection of “c.317G>A (p.C106Y) homozygous pathogenic mutation” in the *SI* gene, sacrosidase enzyme replacement therapy was started with diet. Clinical and symptomatic improvement was observed.

### Pedigree Analysis and Evaluation of Family Members

**Figure 1.**
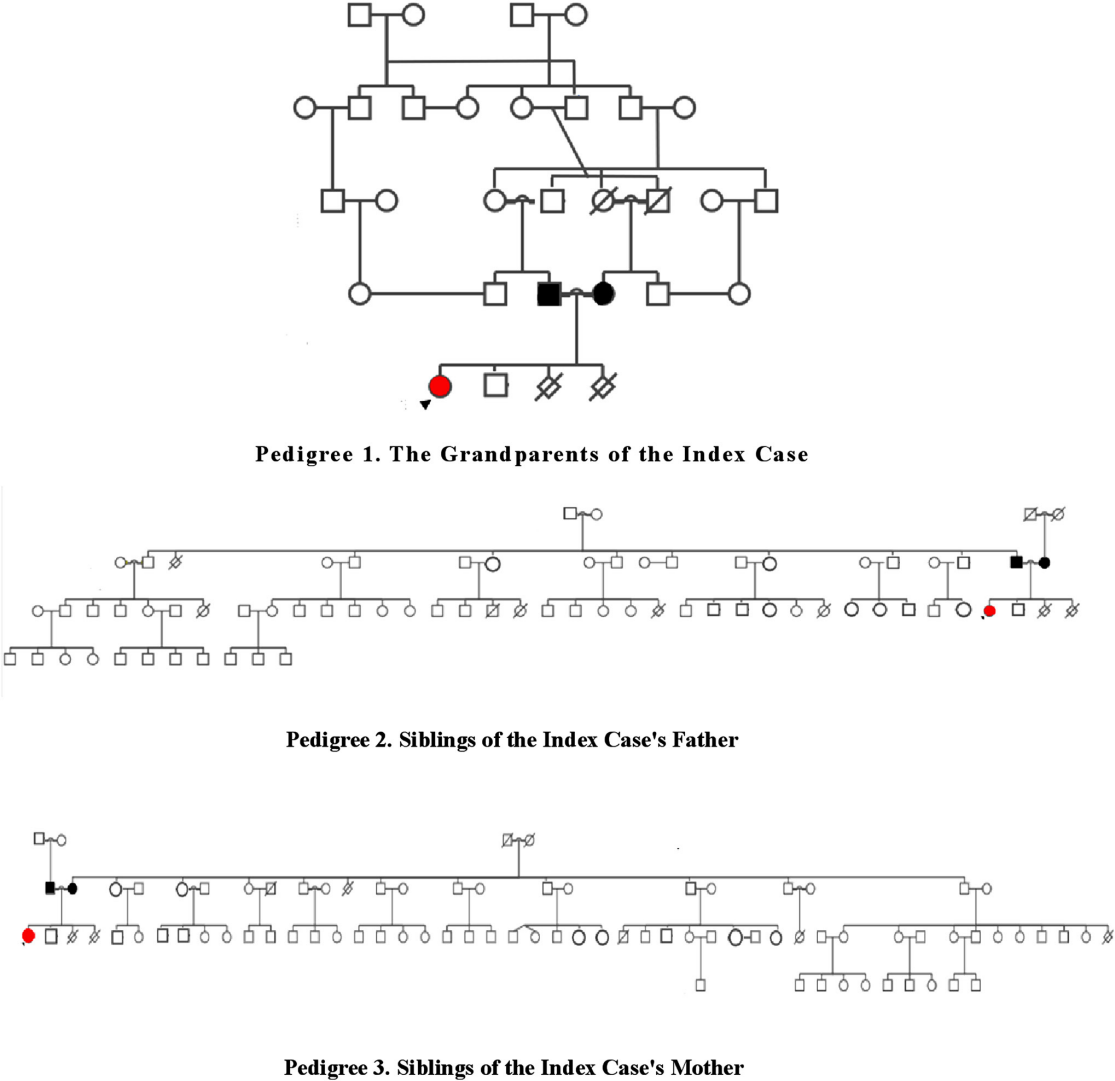
Pedigrees of the index case.

In the family history of the index case, her parents were cousins and her father had chronic diarrhea in childhood. In addition, some family members had consanguineous marriages, and a pedigree analysis was performed (Figure 1). It was a large family and the majority of them lived in the same village. Due to its remote location, which is approximately 520 km from our hospital, it was almost impossible for all family members to visit our clinic. Therefore, our medical team, which included the doctor, nurses, and interviewers, traveled to the settlement where the family resided.

Blood samples were taken from the family members, and their demographic data, detailed nutritional history starting from infancy, complaints, and physical examination findings were recorded. Failure to thrive was defined as weight consistently below the third percentile for age and sex, progressive decrease in weight below the third percentile, or body mass index (BMI) *Z*-score of less than −2 SDs.

### Genetic Analysis

The deoxyribonucleic acid (DNA) isolation protocol was applied to peripheral blood lymphocyte cells in 2 mL blood samples taken from the attendees into an ethylenediaminetetraacetic acid tube. A EZ1 DNA Blood kit for DNA isolation (Catalog No: 951034 QIAGEN, Germany) and an EZ1 Advanced XL Robotic DNA isolation device were used. In the obtained DNA, the exon and exon–intron junctions of the *SI* gene were amplified with primers using a polymerase chain reaction (PCR)-based target enrichment method. The samples were run on a 2% agarose gel to evaluate whether the PCR products were amplified. The GeneDireX PCR Clean-Up & Gel Extraction Kit (Product No: NA006-0300, GeneDireX) was used for the purification of PCR products following gel imaging. A Nextera XT DNA Library Prep Kit (Illumina, San Diego, Calif, USA) was used for the library preparation and the Integrative Genomics Viewer program was used for the evaluation of samples. Taking the human genome “hg38” as a reference, Human Gene Mutation Database (HGMD) and ClinVar databases were used to evaluate the detected variations, and those that were detected for the first time were evaluated using the MutationTaster, PolyPhen-2, SIFT, and VarSome modeling programs.

### Ethics Committee Approval

This study was performed in line with the principles of the Declaration of Helsinki. Ethics committee approval was obtained from Karadeniz Technical University Scientific Researches Ethics Committee (Approval number: 2021/287), and informed voluntary consent was obtained from all family members and/or parents who agreed to participate in the study.

### Statistical Analysis

All statistic evaluations in our study were carried out using IBM Statistical Package for the Social Sciences Statistics (version 23.0. IBM Corp., Armonk, NY, USA). As descriptive statistics, if parametric test assumptions were provided for numerical variables, mean ± SD was used, and median (minimum–maximum) if not; categorical variables are presented in numbers (n) and percentages (%). Categorical data were compared with a chi-square test, and situations in which the *P* < .05 were considered statistically significant.

## Results

Among the 130 family members identified in the pedigree analysis of the index case, 116 were interviewed. Seven individuals declined to participate in the study. Of the 109 participants, 61 (56%) were male, and the mean age was 22.6 ± 17.2 years (0.1-75 years).

Genetic analysis revealed a c.317G>A (p.C106Y) heterozygous mutation in the *SI* gene of 27 cases (24.7%). Fourteen participants (51.9%) were male; the mean age was 23.2 ± 18.3 years (0.1-75 years).

Relevant symptoms or clinical findings were observed in 33 (30.3%) of all participants, including GI symptoms (25%), nephrolithiasis (12.9%), and failure to thrive (6.4%).

Twelve (44.4%) cases with the mutation were symptomatic and had symptoms such as abdominal pain (n = 10, 37%), gas irritability (n = 9, 33.3%), bloating (n = 6, 22.2%), chronic diarrhea (n = 5, 18.5%), and foul-smelling stools (n = 5, 18.5%). Nine (33.3%) cases had 2 or more GI symptoms. Among the other clinical findings, failure to thrive was present in 4 patients (14.9%) and nephrolithiasis was present in 3 patients (17.6%) (Table 1). In the symptomatic cases with the heterozygous mutation, other possible causes of symptoms (celiac disease, food allergies, other GI diseases, etc.) were excluded by biochemical, serological, and endoscopic evaluation.

Symptoms were present in 21 (25.6%) of 82 cases with no mutation. A comparison of the symptomatic cases with and without mutations revealed a significant difference with respect to symptom frequency, sibling death history, and recurrent hospital admissions (*P* = .017, *P* = .002, and *P* = .011, respectively). When compared in terms of symptoms, the frequency of gas irritability, foul-smelling stool, and ≥2 GI symptoms were significantly higher in the group with mutations (*P* = .005, *P* = .047, and *P* = .049, respectively). Postprandial symptoms related to nutrition and food allergy history were significantly higher in cases with mutations (*P* = .017 and *P* = .021, respectively).

Sucrose- and starch-restricted diet were prescribed primarily to the 12 symptomatic cases with no other concomitant disease. Five patients whose symptoms regressed with diet were included in the clinical follow-up. Sacrosidase enzyme replacement therapy (1 mL/meal for children under 15 kg, 2 mL/meal for children over 15 kg) were given in 7 cases unresponsive to diet for 2 months.^1^ After enzyme replacement, regression of GI symptoms was observed in all cases.

**Figure 2.**
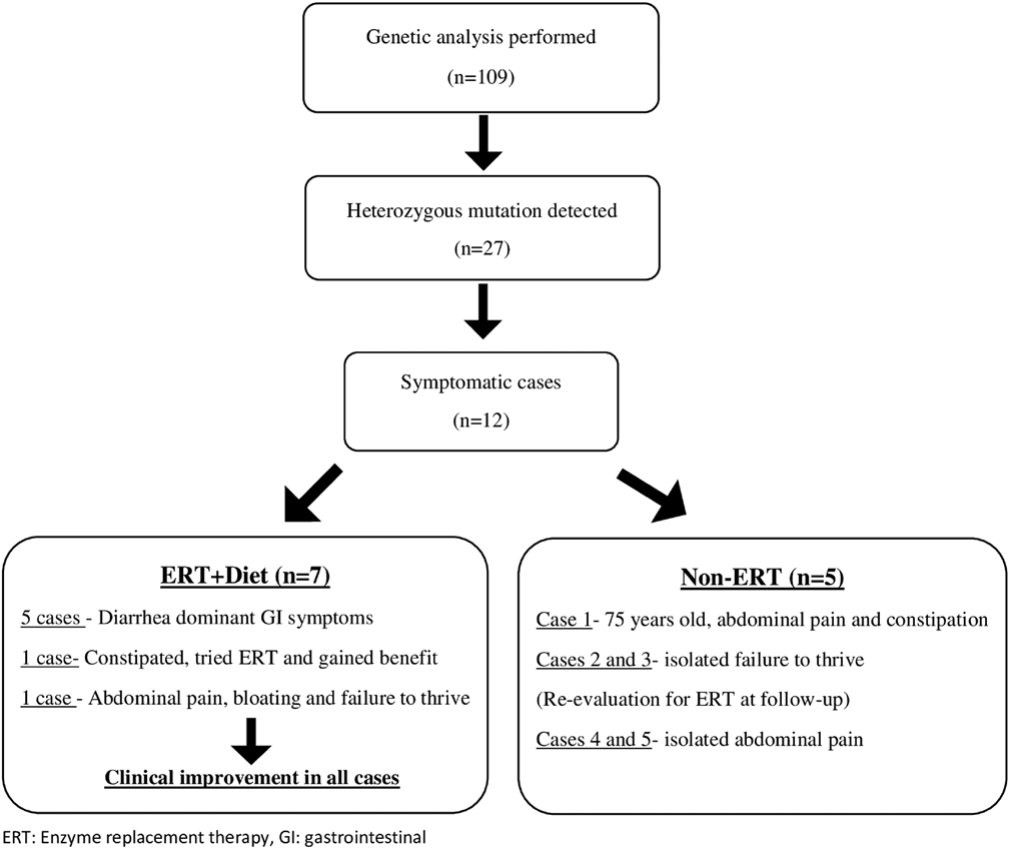
Follow-up of cases with mutations.

Based on their clinical, genetic, and treatment responses, 7 cases (6.4%) with heterozygous mutations were diagnosed with CSID (Figure 2). When the index case with the homozygous mutation was included, the frequency of CSID was determined as 7.3% (8/110).

## Discussion

The current study involved symptom evaluation and *SI* gene mutation analysis in 109 family members of our index case with CSID. It is the first large-scale study in our country with 27 heterozygous cases other than our homozygous index case.

Twelve (44.4%) of the 27 cases with heterozygous mutations were symptomatic, and although they had the same mutation, clinical presentations were different. Therefore, the disease should be evaluated not only by the presence of mutation and the presence of symptoms is multifactorial which depends on age, enzymatic activity level, nutritional status, as stated in the literature.^6^ Despite the AR inheritance pattern of the disease, the presence of symptomatic heterozygous cases, as well as the presentation of different clinical features with the same mutation, provides an opportunity to discuss approaches to diagnosis and treatment.

Various molecular defects have been identified as underlying causes of different clinical scenarios in CSID, such as intracellular glycosylation and folding, intracellular transport, and targeting and insertion of the enzyme into the brush border membrane.^14^ In most cases, both sucrase and isomaltase activities are completely absent. However, in some cases, it was observed that the enzyme was located in the brush border membrane and the mutation only affected the catalytic site of sucrase so that the sucrase activity was absent and the isomaltase activity reduced by 50%-90%.^7^ On the other hand, the sucrase–isomaltase enzyme is responsible for 80% of the degradative activity of maltase, and maltase activity is also reduced in sucrase–isomaltase enzyme deficiency.^15^ Eggermont and Hers^16^ reported that there is a structural relationship between sucrase–isomaltase and maltase–glucoamylase, and an abnormality in any of them affects both enzymes.

Although symptoms are mostly defined in homozygous or compound heterozygous cases, they have also been reported in heterozygous individuals diagnosed with irritable bowel syndrome (IBS) in recent years.^17^ In heterozygous patients, chronic abdominal pain predominates rather than osmotic diarrhea, which is commonly observed in homozygous patients. Other GI findings include chronic dyspepsia and nausea and vomiting.^18^ Deb et al^19^ reported that the frequency of heterozygous mutations in the *SI* gene in cases with IBS-like symptoms was 3.5%, while symptomatic heterozygous cases with abnormal sucrase activity were reported as 23.2%. Although classical homozygous CSID is generally similar to diarrhea-predominant IBS, Henström et al^20^ reported constipation-predominant or mixed-type IBS in heterozygous CSID variants. In our study, the most common GI complaints that were reported in 10 (37%) of the 27 patients with heterozygous mutations were abdominal pain and gas irritability. While 1 of our cases had recurrent hospital admissions due to chronic constipation, laboratory tests and colonoscopy were normal. The patient, who did not respond to medical treatment due to chronic constipation, benefited from sacrosidase treatment. Enzyme replacement therapy was not planned for our patient at the beginning, but the clinical response after the sacrosidase trial treatment suggested that constipation may also be one of the treatment indications.

Concurrent CSID and nephrolithiasis was first described in 1970, though the pathogenesis was not clarified.^21^ Belmont et al^22^ claimed that sucrase–isomaltase and lactase play a role in intestinal calcium homeostasis and that in the event of deficiency, nephrocalcinosis secondary to hypercalcemia may occur with the upregulation of the 1,25-OH-VitD3 receptor. In addition, it was emphasized that CSID should be kept in mind, particularly in infantile hypercalcemia with failure to thrive and a history of chronic diarrhea. Although the data on the coexistence of nephrolithiasis and CSID are limited,^21-23^ the father of the index case had a history of nephrectomy due to nephrolithiasis before diagnosis. Therefore, urinary system ultrasonography and complete urinalysis were planned for the cases with mutations. Nephrolithiasis was detected in 3 of the heterozygous cases and in our index case. However, since urinary system radiological examination could not be performed in all cases, the data could not be compared statistically.

Data on food allergies in enzyme deficiencies such as CSID are insufficient, and mostly food sensitivities and formula intolerance have been reported.^24^ The inability to digest sucrose and starch in the diet leads to the induction of symptoms.^25^ In our study, the history of food allergy was significantly higher in cases with the mutation; however, when the allergy history was reviewed, it was noticed that the families attributed symptoms such as diarrhea or the development of a rash after feeding to food allergies, without confirmation using skin prick tests. We think that this situation is related to food intolerance rather than allergy.

The gold standard diagnosis of the disease is based on SI enzyme activity in small intestinal mucosa samples. Among the noninvasive tests, the sucrose challenge test or ^13^C-sucrose breath test can also help in diagnosis.^26^ However, ^13^C-sucrose breath test or sucrase–isomaltase enzyme activity measurements cannot be performed in Turkey. In the study by Karakoyun et al,^27^ 5 cases were diagnosed based on a sucrose challenge test following a detailed nutritional history and the determination of the relationship between symptoms. Although “*SI* gene” mutation analysis, which is another diagnostic method, is not primarily preferred due to its high cost, it has been used in Turkey in recent years.^28^ Although the pathogenic effect of the homozygous mutation in our index case could not be proved by enzyme activity, it was confirmed by clinical improvement after sacrosidase enzyme replacement and sucrose–starch-restricted diet.

Treatment involves a lifelong sucrose- and starch-restricted diet adapted to the requirements of the patient. However, the presence of sucrose and starch in frequently consumed foods such as potatoes, onions, tomatoes, and green beans, as well as many fruits, makes dietary compliance difficult. In this case, it has been shown that sacrosidase enzyme replacement therapy is much more effective, and the quality of life and nutrition of patients improve significantly.^29^ Enzyme replacement therapy was initiated in our index case and in 7 patients with heterozygous mutations with prominent symptoms and we obtained a good response in all of them. Other cases were followed up with a sucrose- and starch-restricted diet. Although the presence of GI symptoms unresponsive to diet, failure to thrive, and/or nephrolithiasis are considered as primary indications for initiation of therapy, we also had a case whose chronic constipation regressed after a 10-day trial period. This observation shows that further studies are needed to determine the correct treatment indications, especially in heterozygous cases. Enzyme replacement therapy is not suitable for all patients with heterozygous mutations due to its high cost. In cases where it is not feasible to study the enzymatic activity level, such as those in Turkey, evaluating the clinical response to short-term enzyme use can be an appropriate treatment decision, although it is not cost-effective.^30^ We think that more studies are needed to make the diagnosis more practical and cost-effective, especially in heterozygous cases, and to clarify the treatment indications.

In conclusion, the diagnosis of CSID, which is known to have an AR inheritance, is often missed due to the diversity of clinical symptoms, diagnostic difficulties, and lack of awareness. We think that further studies are needed to evaluate the clinical management, indications for enzyme replacement therapy, and the genotype–phenotype relationship in patients with heterozygous mutations.

## Figures and Tables

**Table 1.** Comparison of Cases with and Without Heterozygous Mutations

Parameters (%)	Mutation (+)	Mutation (−)	*P*
	(n = 27)	(n = 82)	
Presence of symptoms	44.4	25.6	.056^*^
Abdominal pain	37	23	.075
Bloating	22.2	12.8	.147
Gas irritability	33.3	10	.005^*^
Chronic diarrhea	18.5	10	.160
Foul-smelling stools	18.5	5.7	.047^*^
Constipation	14.9	10	.363
≥2 GI symptoms	33	15.8	.049^*^
Failure to thrive	14.9	3.65	.062
Nephrolithiasis	17.6	11.7	.382
Diaper dermatitis	3.7	2.9	.605
Nutrition
		
Postprandial symptoms	18.5	2.8	.017^*^
Fruit consumption	88.8	91.2	.499
Avoiding sweet foods	37	22	.090
Food allergy	11	1.5	.021^*^
Hospital admission	26	2.8	.002^*^
Consanguinity	51.8	50	.526
Sibling death	66.6	38.2	.011^*^

^*^ *P* <.05.
